# Fc-mediated functions and the treatment of severe respiratory viral infections with passive immunotherapy – a balancing act

**DOI:** 10.3389/fimmu.2023.1307398

**Published:** 2023-11-22

**Authors:** Hillary A. Vanderven, Stephen J. Kent

**Affiliations:** ^1^ Biomedical Sciences and Molecular Biology, College of Public Health, Medical and Veterinary Sciences, James Cook University, Douglas, QLD, Australia; ^2^ Australian Institute of Tropical Health and Medicine, James Cook University, Douglas, QLD, Australia; ^3^ Department of Microbiology and Immunology, Peter Doherty Institute for Infection and Immunity, University of Melbourne, Parkville, VIC, Australia; ^4^ Melbourne Sexual Health Centre and Department of Infectious Diseases, Alfred Health, Central Clinical School, Monash University, Carlton, VIC, Australia

**Keywords:** antibody-based therapy, passive immunotherapy, influenza virus, SARS-CoV-2, respiratory syncytial virus (RSV), antibody-dependent cellular cytotoxicity, natural killer cells

## Abstract

Passive immunotherapies have been used to treat severe respiratory infections for over a century, with convalescent blood products from recovered individuals given to patients with influenza-related pneumonia as long ago as the Spanish flu pandemic. However, passive immunotherapy with convalescent plasma or hyperimmune intravenous immunoglobulin (hIVIG) has not provided unequivocal evidence of a clinical benefit for severe respiratory infections including influenza and COVID-19. Efficacy trials, primarily conducted in late-stage disease, have demonstrated inconsistent efficacy and clinical benefit for hIVIG treatment of severe respiratory infections. To date, most serological analyses of convalescent plasma and hIVIG trial samples have focused on the measurement of neutralizing antibody titres. There is, however, increasing evidence that baseline antibody levels and extra-neutralizing antibody functions influence the outcome of passive immunotherapy in humans. In this perspective, findings from convalescent plasma and hIVIG trials for severe influenza, COVID-19 and respiratory syncytial virus (RSV) will be described. Clinical trial results will be discussed in the context of the potential beneficial and deleterious roles of antibodies with Fc-mediated effector functions, with a focus on natural killer cells and antibody-dependent cellular cytotoxicity. Overall, we postulate that treating respiratory viral infections with hIVIG represents a delicate balance between protection and immunopathology.

## Introduction

The field of passive immunisation, or transfer of antibody to confer temporary protection, began in 1890 when Behring and Kitasato showed that a rabbit could be protected from tetanus by injecting serum from an immunized animal (1, 2). This approach is applied to protect humans against life-threatening bacterial toxins (e.g. diphtheria) and for pre- and post-exposure prophylaxis against viral infections (e.g. rabies, hepatitis A and B), but it has shown mixed results for treatment of severe viral infections. Products for passive immunotherapy include convalescent plasma (CP), intravenous immunoglobulin (IVIG), hyperimmune IVIG (hIVIG) and monoclonal antibodies (mAbs) (3). CP is pooled from blood of patients who have recovered from infection. Both IVIG and hIVIG are concentrated immunoglobulin G (IgG) prepared from pooled human plasma. IVIG is derived from plasma pooled from thousands of healthy donors, whereas hIVIG is made with plasma pooled from donors with high levels of pathogen-specific antibody (recovered or recently vaccinated). Treatment with hIVIG presents several advantages over CP including control over antibody concentration and lower infusion volume. However, CP can be produced more quickly than hIVIG, which is desirable during a pandemic (2, 4). Passive immunotherapy for viral infections introduces antibodies with direct and indirect anti-viral functions to clear virus and infected cells.

**Figure 1:**
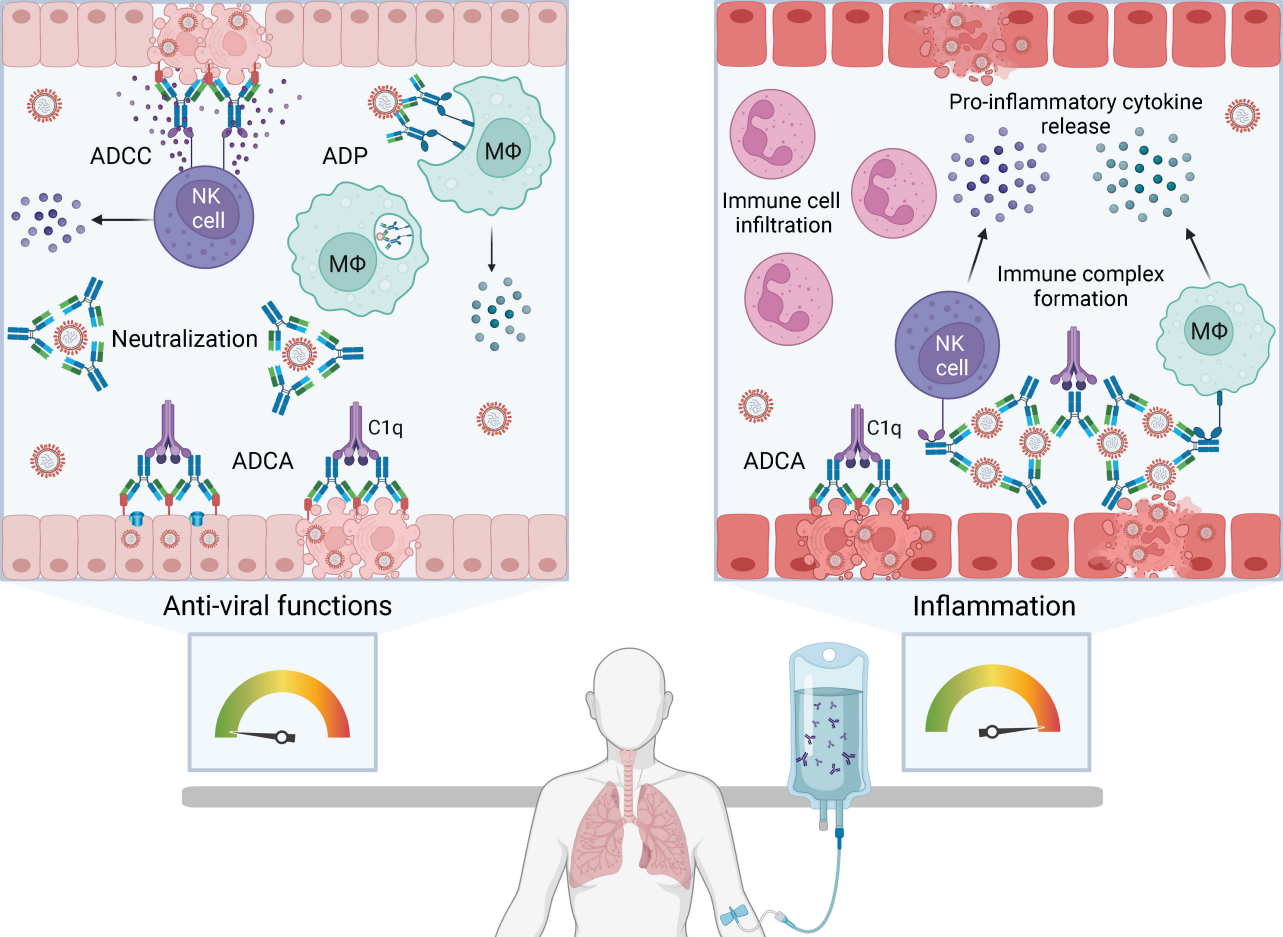
The balance between anti-viral functions and inflammation with passive immunotherapy for severe human respiratory viral infections. Passive immunotherapies introduce exogenous antibody that can mediate an array of protective anti-viral functions (left box) but also have the potential to cause immunopathology by enhancing pathological inflammation (right box). Antibody-based therapies can directly neutralize extracellular virus and induce Fc-mediated functions including antibody-dependent cellular cytotoxicity (ADCC), antibody-dependent phagocytosis (ADP) and antibody-dependent complement activation (ADCA). ADCC is initiated by FcγRIIIa on natural killer (NK) cells or macrophages (Mϕ) engaging Fc domains of immunoglobulin G (IgG) bound to viral antigens on infected cells. Multimeric engagement of FcγRIIIa on NK cells triggers release of cytolytic granules resulting in apoptosis of virus-infected cells and release of pro-inflammatory cytokines and chemokines, including interferon gamma (IFNγ) and macrophage inflammatory proteins (MIP1α and MIP1β). ADP is the uptake of antibody-opsonised virus or infected cells by phagocytes expressing FcγRI and FcγRIIa. ADCA is induced when C1q binds to the Fc domain of antibodies bound to virions or virus-infected cells, which can lead to lysis via membrane attack complex insertion. Formation and deposition of immune complexes can drive inflammation through FcγR-dependent activation of immune cells, such as NK cells, macrophages and neutrophils, leading to the production of high levels of pro-inflammatory cytokines. Immune complexes can also initiate ADCA resulting in anaphylatoxin release and leukocyte infiltration. Created with BioRender.com.

Neutralization of virus by antibody fragment antigen-binding (Fab) domains prevents infection of cells by blocking viral attachment, fusion and entry. Blood products used in passive immunotherapy are rich in IgG, the major antibody isotype found in human plasma. The IgG fragment crystallizable (Fc) domain selectively binds to activating or inhibitory Fc gamma receptors (FcγR) on immune effector cells inducing anti-viral functions such as antibody-dependent cellular cytotoxicity (ADCC) and antibody dependent phagocytosis (ADP). Fc-mediated functions may be especially important in passive immunotherapy to promote clearance of virus-infected cells and extracellular virus in an infected host (**Figure 1**, left box). However, antibody Fc functions can also result in antibody-dependent enhancement (ADE) of viral infection or disease.

Two mechanisms of ADE have been described: extrinsic and intrinsic. Extrinsic ADE increases viral entry into phagocytes through FcγRIIa-dependent uptake of antibody-opsonised virus. Non-neutralizing antibody binds virus (e.g. dengue) shuttling it into macrophages that become productively infected (5–7). Intrinsic ADE involves Fc-mediated functions triggering a strong inflammatory response (8). An example of intrinsic ADE is the formation of immune complexes from non-neutralizing antibodies, leading to Fc-mediated activation of immune cells and complement resulting in anaphylatoxin release and leukocyte infiltration (**Figure 1**, right box) (6, 9, 10). Intrinsic ADE can promote inflammation in the respiratory tract, airway obstruction and in serious cases, acute respiratory distress syndrome (ARDS) (9–12). Whether passive immunotherapy contributes to intrinsic ADE during viral infection is unclear. IVIG has immunomodulatory and anti-inflammatory properties used to treat autoimmune and inflammatory disorders. It is postulated that CP and hIVIG may have immunomodulatory effects that blunt inflammation (13), but this is not well understood.

A systematic review and meta-analysis of the effectiveness of convalescent blood products for severe influenza and severe acute respiratory syndrome (SARS) concluded that CP may reduce mortality, but well-designed trials were necessary to establish efficacy (14). Without compelling evidence of efficacy, the high cost of treating viral infections with passive immunotherapy may present a significant limitation to its clinical use. Herein, we describe findings from CP and hIVIG trials for influenza, COVID-19 and respiratory syncytial virus (RSV) relating to potential beneficial and harmful roles for Fc-mediated antibody functions. We also highlight possible contributions of ADCC and natural killer (NK) cells to the equilibrium between protection and pathology when using passive immunotherapy to treat respiratory viruses.

## Influenza virus

### Influenza-convalescent plasma therapy

Historical studies show that passive immunotherapy with convalescent blood products may be an effective treatment for severe influenza. During the Spanish flu pandemic, influenza-convalescent blood products were reported to reduce morbidity and mortality in patients with influenza-associated pneumonia and ARDS (15). A meta-analysis of 8 historical studies and 1703 patients examined the impact of transfusing influenza-convalescent whole blood, plasma or serum on mortality from Spanish influenza complicated by pneumonia (16). The overall crude case-fatality rate was lower for treated patients (16%) compared to untreated controls (37%; p < 0.001). Patients who received treatment earlier, within 4 days of pneumonia, had reduced overall crude case-fatality rates (19%) relative to patients who received later treatment (59%; p <0.001) (16). This subgroup analysis highlights that timing of treatment is critical. When administered early in the course of infection convalescent blood products reduced fatalities, but if received in later stages of disease, case-fatality rates were higher than in untreated controls. CP therapy also decreased respiratory tract viral load, serum cytokine levels and mortality (20% treated vs. 54.8% untreated; p = 0.01) in patients with severe pandemic H1N1 2009 (H1N1pdm09) influenza (17). Convalescent blood products reduced mortality from pandemic H1N1 influenza (16, 17), if given early in the course of disease, but the mechanisms underpinning this survival benefit remain largely unexplored.

A more recent randomised phase II clinical trial (n=98) found that immune plasma [hemagglutination inhibition (HAI)≥80] was safe but did not show a clear treatment benefit for severe influenza A or B based on respiratory normalisation by day 28 (18). A larger phase III clinical trial found that high-titre immune plasma (HAI≥80) conferred no benefit over low-titre plasma (HAI ≤ 10) for severe seasonal influenza A, demonstrating that HAI titre alone does not determine clinical efficacy of CP (19). In both trials, participants had a median of 3-4 days of influenza illness before enrolment. Extra-neutralizing Fc functions, including NK cell activation and ADCC, were not measured in CP trials and their impact on efficacy is unknown.

### Hyperimmune intravenous immunoglobulin therapy for severe influenza

Inconclusive results for CP led to renewed interest in hIVIG therapy for severe influenza. A small (n=35) randomised controlled trial compared hIVIG, prepared from H1N1pdm09 CP, to IVIG prepared before 2009 for hospitalised adults with influenza (4). The hIVIG had high neutralizing antibody (NAb) titres (≥640) against the H1N1pdm09 virus, and IVIG had no detectable NAbs against this virus. Respiratory viral loads were lower in the hIVIG treated group at days 5 and 7 post-infusion, but mortality and length of hospital stay were not significantly different between groups (4). A subgroup analysis of subjects who received hIVIG or IVIG within 5 days of symptom onset showed that hIVIG was the only factor that independently reduced mortality. In contrast, all 5 subjects who received hIVIG later than 5 days after symptom onset died, while all 7 subjects treated with IVIG at this later timepoint survived. This demonstrated that early administration of hIVIG conferred a survival benefit but introduced the possibility of deleterious effects if given later in the course of influenza. Investigators proposed that hIVIG had anti-viral and immunomodulatory activities including neutralization, ADCC, blocking of IL-1a-dependent leukocyte recruitment and decreased cytokine mRNA levels. However, the high mortality rate in patients who received hIVIG late in the course of disease, and possible underlying mechanisms, were not addressed.

A phase III clinical trial (n=308) used a hIVIG called Flu-IVIG to treat patients hospitalised with severe seasonal influenza A or B within 7 days of symptom onset (20). The Flu-IVIG had no clinical benefit for patients with severe influenza A, despite causing a large rise in HAI titre. However, Flu-IVIG showed a significant treatment benefit for influenza B. Flu-IVIG antibodies targeting influenza B virus had a higher affinity than antibodies against influenza A virus, leading to slower dissociation of immune complexes (20). Since immune complex functions are driven by interactions with FcγRs on effector cells, we undertook a detailed serological analysis of Flu-IVIG clinical samples to measure extra-neutralizing antibody Fc functions (21). We showed that influenza A-specific FcγRIIa- and FcγRIIIa-binding antibody, as well as antibody-dependent NK cell activation, were associated with worse outcomes in patients with severe seasonal influenza A at day 5 and 7 post-infusion. Flu-IVIG also worsened the odds of a favourable clinical outcome in influenza A patients with low pre-infusion levels of FcγR-binding antibody compared to placebo. The opposite effect was observed for severe influenza B, with a trend towards higher influenza B-specific FcγR-binding antibody being associated with better outcomes and Flu-IVIG therapy improving the odds of a favourable outcome in patients with low FcγR-binding antibody at baseline (21). Overall, Fc-mediated antibody functions appeared to be either deleterious or beneficial depending on the infecting influenza virus genotype and host immune factors including pre-existing immunity.

A growing body of work supports a potential protective role for influenza-specific ADCC (22–26), but pre-clinical and observational studies suggest that Fc-mediated functions can also cause immunopathology. Humans with more severe influenza symptoms have higher HA-specific ADCC activity (25, 27). Elevated levels of non-neutralizing antibodies and pathogenic immune complexes were identified in patients with severe and fatal infections with H1N1pdm09 virus (28, 29). Mice vaccinated with an ADCC epitope had increased perforin and inflammatory cytokine levels in the lungs, greater alveolar damage and higher mortality following H1N1pdm09 infection compared with control mice (8), suggesting that ADCC may increase pulmonary inflammation and leukocyte infiltration. A modest decrease in viral load was observed in vaccinated mice, but any protective effect was outweighed by inflammatory lung damage. Higher mortality with late administration of influenza-CP or hIVIG was independently reported across trials and meta-analyses (4, 16). Exogenous antibody introduced late in the course of influenza illness, after an endogenous antibody response has been mounted and virus replication is decreasing, may promote formation of inflammatory immune complexes. In this case, Fc-mediated functions could be detrimental by enhancing immune cell activation and inflammation without providing any significant benefit for viral clearance. Findings from the Flu-IVIG trial indicate that hIVIG may not be an effective therapy for influenza A, given the association between FcγR-binding antibody and poorer outcomes (20, 21). Conversely, hIVIG demonstrated a treatment benefit for influenza B and larger trials should be undertaken. Paradoxical hIVIG results show that a complex balance between Fc-mediated anti-viral functions and pathological inflammation may exist when treating severe influenza with passive immunotherapies.

## Severe acute respiratory syndrome coronavirus 2

### COVID-19 convalescent plasma therapy

CP therapies were tested in subjects with mild and severe COVID-19, with few showing any clinical benefit (30–37). Transfusion of CP with high titres of anti-SARS-CoV-2 IgG decreased the risk of death in COVID-19 patients hospitalised without mechanical ventilation, compared to patients who received low-titre plasma (32). Another trial reported a treatment benefit for CP in preventing progression to death and ventilation in patients hospitalised with COVID-19 at 28 days post-infusion (36). Conversely, large randomised controlled trials of COVID-19 CP showed no difference in mortality or other measures of outcome between treated and control groups (33–35, 37), with the caveat that treatment occurred late in the course of disease (median 8-9 days after symptom onset). Therapeutic mAbs were effective against pre-Omicron SARS-CoV-2 variants when administered early, but not late, in the course of disease. A recent study showed that NAb titre is a key determinant of therapeutic efficacy, but CP and hIVIG fell below the threshold for high level protection (38, 39). CP therapy failed to provide a clear clinical benefit for COVID-19 but yielded important insights into potential protective mechanisms.

Anti-SARS-CoV-2 spike protein (S) IgG and NAb titre were the main serological parameters measured in COVID-19 CP trials, but ADCC and antibody-dependent NK cell activation were explored. The CP for COVID-19 Respiratory Illness (CONCOR-1) trial found that antibody content of CP modified its therapeutic effect. High ADCC activity in CP was independently associated with lower risk of intubation and death 30 days post-infusion (35), suggesting that enhanced ADCC may provide a therapeutic benefit. This is consistent with findings in animal models and severe COVID-19 cohorts that showed a protective role for Fc-mediated functions in SARS-CoV-2 infection (40–45). A small (n=80) study showed that CP blunted inflammatory S-specific antibody features, like C1q- and FcγRIIa-binding, while enhancing nucleocapsid (N)-specific antibody responses (13). Patients who received the greatest benefit from COVID-19 CP therapy had the lowest baseline Fc-functionality, including low ADP and antibody-dependent NK cell macrophage inflammatory protein 1β (MIP1β) production. Interestingly, N-specific antibody-dependent NK cell activation and MIP1β production were the features most strongly associated with better clinical outcomes, despite not being enriched in the treatment group. Ullah et al. showed that robust Fc functions in CP protected mice from lethal SARS-CoV-2 infection (46). Together, these findings suggest that CP efficacy against COVID-19 may partially depend on Fc-mediated functions like NK cell activation and ADCC.

### Hyperimmune intravenous immunoglobulin therapy for severe COVID-19

Standardised hIVIG preparations with higher concentrations of anti-SARS-CoV-2 antibody may overcome CP variability and improve therapeutic efficacy. A small trial reported that COVID-19 hIVIG was safe, increased survival and reduced the risk of disease progression in subjects severely or critically ill with ARDS (47). A phase III clinical trial (n=593) of hIVIG showed no efficacy in patients hospitalised with COVID-19 without end-stage organ failure. The hIVIG was administered late in the course of infection, a median of 8 days after symptom onset (48). Interestingly, hIVIG safety was altered by the presence of endogenous SARS-CoV-2 antibody at randomisation. In the hIVIG group, there was an increased risk of adverse events at day 7 post-infusion for subjects who were NAb-positive at baseline, compared to those who were NAb-negative. The underlying cause of adverse events in hIVIG-treated NAb-positive subjects remains to be elucidated, but hIVIG may be harmful in certain subgroups of hospitalised COVID-19 patients and beneficial in others (48), as we observed for influenza (21). This highlights the need to analyse FcγR-binding and ADCC in these clinical samples to uncover associations with outcome.

Antibody-dependent NK cell activation and ADCC have been linked to SARS-CoV-2 protection in CP trials and cohort studies (35, 44, 45), but Fc-mediated functions may drive inflammation in severe COVID-19. Antibody features, such as S-specific C1q- and FcγRIIa-binding, were associated with more severe disease (13). Antibody-dependent complement activation is enhanced in patients with severe COVID-19 and may increase inflammatory lung injury (49, 50). FcγR-dependent uptake of SARS-CoV-2 by macrophages/monocytes results in inflammatory cell death and systemic inflammation (51), and FcγR-mediated activation of monocytes by anti-SARS-CoV-2 IgG promotes tumer necrosis factor alpha (TNFα) release (49). Severe COVID-19 is associated with an afucosylated anti-S antibody signature (52, 53). Afucosylated IgG has a high affinity for FcγRIIIa and is a potent mediator of ADCC, indicating that antibody-dependent NK cell activation and ADCC may be exacerbating COVID-19 disease. Patients with worse COVID-19 symptoms have elevated serum ADCC activity, and the high affinity variant of FcγRIIIa (158-V/V) is overrepresented in hospitalised and deceased COVID-19 patients (54, 55). There is no unequivocal evidence of ADE of human SARS-CoV-2 infection, but immune complexes formed with inflammatory anti-S antibodies may over-activate complement and immune cells by binding to C1q and FcγRs, contributing to COVID-19 pathogenesis in some individuals (56).

Collectively, CP and hIVIG have not demonstrated significant impact on COVID-19 mortality or clinical outcome. However, these trials revealed key information about protective and pathological features of SARS-CoV-2 antibodies. Further insights can be gained into the balance of harmful and beneficial antibody profiles in COVID-19 by measuring antibody Fc-functionality in hIVIG trials.

## Respiratory syncytial virus

### Immunoprophylactic antibodies against RSV

A hIVIG (RSV-IVIG) and mAbs against the RSV fusion (F) protein (palivizumab and nirsevimab) have been approved for prophylactic use against RSV in high-risk infants and children (57–59). RSV-IVIG was marketed from 1996-2003 for RSV prevention in children younger than 2 years of age with a history of prematurity or chronic lung disease (60). Combination therapy with RSV-IVIG and ribavirin improved outcomes in bone marrow transplant recipients and reduced lung viral load in RSV-infected cotton rats compared to ribavirin alone (61, 62). Prophylactic administration of RSV-IVIG to children and infants with bronchopulmonary dysplasia and/or prematurity reduced the incidence of lower respiratory tract infections and hospitalisations (63, 64), but there is no conclusive evidence to support a role for RSV-IVIG in treatment of severe RSV (65, 66). RSV-IVIG was prone to unpredictable efficacy profiles and was replaced by palivizumab (58), which neutralizes RSV with a potency ~50 times greater than RSV-IVIG (60, 67). Palivizumab and nirsevimab are administered intramuscularly and indicated for prophylaxis only (58, 59).

The Fc-mediated functions of RSV-IVIG have not been assessed, but detection of RSV-specific ADCC in the respiratory tract of infants during natural infection was first reported over 40 years ago (68). RSV-specific ADCC activity was independent of symptom severity and waned rapidly compared to neutralization (68, 69). Antibodies targeting RSV attachment (G) protein are more efficient inducers of ADCC compared to anti-RSV F protein antibodies (70, 71). Evidence of a protective role for ADCC in animal models of RSV is limited. Fab fragments derived from anti-RSV G protein mAbs did not reduce viral load in RSV-infected mice, but corresponding mAbs with intact Fc domains conferred protection (72).

In the 1960s, a formalin-inactivated RSV vaccine caused vaccine-induced ADE in children (73, 74). Children were exposed to RSV infection shortly after vaccination and developed enhanced respiratory disease (ERD), which was fatal in two cases (73, 75). Follow-up studies revealed that non-neutralizing antibodies formed immune complexes that deposited in the airway activating complement and pro-inflammatory cytokine release (9, 10). Serum antibody and anti-RSV mAbs showed ADE of RSV infection in FcγR-expressing monocytic cell lines (76–78), and binding to FcγRs appeared to reduce neutralizing activity of anti-RSV mAbs except palivizumab (79). RSV-IVIG did not lead to reported cases of ERD, but a history of RSV vaccine-induced ADE is damaging. Future antibody therapies or vaccines for RSV must be evaluated for harmful Fc-mediated effects and characterised to identify protective and inflammatory antibody features.

## Conclusions

Prophylactic use of NAbs is efficacious for targeted protection of at-risk individuals, but antibody-based therapies are being widely tested for treatment of existing viral infections. Passive immunotherapy for severe respiratory virus infection is most effective if administered early in the course of disease, within 4-5 days of symptom onset. At early stages of infection, infused antibodies may enhance anti-viral activity, including neutralization and Fc functions, to clear virus and infected cells. In late-stage disease, Fc-mediated functions may contribute to inflammation outweighing any beneficial effects from viral clearance. High NAb titre correlates with therapeutic efficacy, but the level of Fc functions required for protection with CP or hIVIG remains uncharacterised. Moving forward, in-depth serological analyses, along with other measures of immune function including T cell-mediated immunity, are required to dissect the utility or harm of Fc-mediated antibody functions when treatment is received relatively late in the course of disease.

## Data availability statement

The original contributions presented in the study are included in the article/supplementary material. Further inquiries can be directed to the corresponding author.

## Author contributions

HV: Conceptualization, Writing – original draft. SK: Conceptualization, Writing – review & editing.
